# Retained bullets: to remove or not to remove? Lessons from two clinical scenarios with intracardiac bullet embolization

**DOI:** 10.1093/jscr/rjae584

**Published:** 2024-09-18

**Authors:** Jared B Hinton, Hunter J Landwehr, Andrew M Loudon, Matthew L Moorman

**Affiliations:** Northeast Ohio Medical University, College of Medicine, 4209 St., OH-44, Rootstown, OH 44272, United States; Northeast Ohio Medical University, College of Medicine, 4209 St., OH-44, Rootstown, OH 44272, United States; Northeast Ohio Medical University, College of Medicine, 4209 St., OH-44, Rootstown, OH 44272, United States; Department of Surgery, University Hospitals, 11100 Euclid Ave., Cleveland, OH 44106, United States; Case Western Reserve University, 9501 Euclid Ave., Cleveland, OH 44106, United States; Northeast Ohio Medical University, College of Medicine, 4209 St., OH-44, Rootstown, OH 44272, United States; Department of Surgery, University Hospitals, 11100 Euclid Ave., Cleveland, OH 44106, United States; Case Western Reserve University, 9501 Euclid Ave., Cleveland, OH 44106, United States

**Keywords:** firearm injuries, gunshot wounds, retained bullets, bullet embolization, trauma surgery

## Abstract

Firearm-related injuries in the USA are increasing, with over 105,000 cases annually. Gunshot wounds (GSWs), especially those involving retained bullets, present complex challenges due to bullet trajectories and embolization risks. This study reviews two cases of bullet emboli, focusing on bullet localization strategies and timing of removal. Imaging techniques such as chest X-ray, CT scan, intraoperative fluoroscopy, and transesophageal echocardiogram were employed for localization. In Case 1, a stable patient with a left-back GSW had a bullet embolism from the inferior vena cava to the right ventricle, necessitating prompt removal. In Case 2, an unstable patient with thoracoabdominal GSWs experienced a delayed embolism to the aortic root, requiring multiple surgeries. Effective management of retained bullets involves diverse imaging and timely surgical intervention, especially for stable patients, emphasizing individualized and proactive strategies to enhance outcomes in bullet embolization cases.

## Introduction

Firearm-related injuries are on the rise, with more Americans dying from gun-related injuries in 2021 than any other year recorded [1]. Over 105,000 firearm injuries are reported each year in the USA [2]. As a surgeon, managing these gunshot wounds (GSWs) requires analysis of the bullet trajectory, using the entrance and exit wounds as clues for the potential organs injured [3]. However, when dealing with retained bullets, this process becomes considerably more challenging. Moreover, the rare occurrence of further embolization of the retained bullet, with an incidence of 0.3%, adds another layer of complexity to the management of these cases [2]. Addressing the challenges of locating and treating retained GSWs involves navigating the complex dynamics of bullet behavior upon impact and within the body. Unlike exiting projectiles, retained bullets transfer their remaining kinetic energy to the tissue encountered, potentially causing tissue destruction beyond the bullet’s caliber [3]. For surgeons, the localization of retained projectiles without clear exit wounds or trajectories complicates surgical decision-making and introduces ambiguity in accurately determining the extent of tissue damage. Consequently, the infrequency of such cases, the prioritization of life-threatening injuries, and under-recognition can lead to delays in diagnosis.

Symptomatic bullet emboli are usually diagnosed immediately, but no guidelines exist for their management. Arterial and venous emboli present varied risks and symptoms, influencing the choice of surgical intervention. Arterial emboli present with organ ischemia and are symptomatic in 80% of cases. Prompt retrieval and reperfusion of tissue is performed on diagnosis. In contrast, venous emboli are symptomatic in only 33% of cases. These emboli are often diagnosed after initial presentation and are more often observed [4–6]. Similar to symptomatic bullet emboli, no guidelines exist for their management.

Conservative management for asymptomatic patients is often recommended but may carry an underrecognized risk of harm. Shannon et al. reviewed the success of conservative management in 51 asymptomatic patients with venous bullet emboli. They found that 25% of patients developed complications and three of the observed patients died after conservative management [7].

Intravascular bullet embolization is a rare and unique challenge in trauma surgery. We present two cases of bullet emboli to discuss bullet localization strategy and decision-making for the timing of bullet removal. These cases highlight the importance of locating GSWs, determining bullet trajectories, and locating retained projectiles. These tasks can be more difficult in patients who are hemodynamically unstable, sustain multiple bullets trajectories, or have chronic retained bullets [3].

## Case 1

A 20-year-old male presented with a GSW to the right flank at the L1 vertebral level. The patient complained of abdominal pain, was moving all extremities, was hemodynamically stable, and GCS 15. Chest X-ray detected a bullet overlying the cardiac silhouette. The patient underwent a CT scan of the chest, abdomen, and pelvis with IV contrast, demonstrating hemoperitoneum, grade IV right renal laceration, grade V liver laceration, and bullet overlying the right ventricle with streak artifact. The CT scan did not show pericardial effusion, or hemothorax. The patient was taken emergently to the OR.

**Figure 1:**
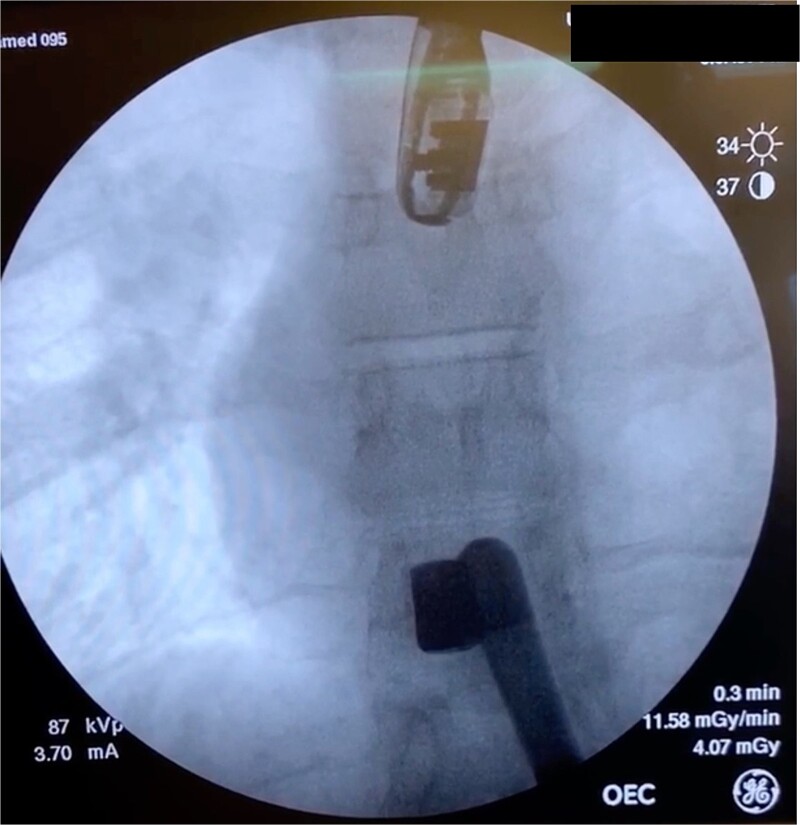
Intraoperative fluoroscopy demonstrating bullet in the right ventricle.

**Figure 2:**
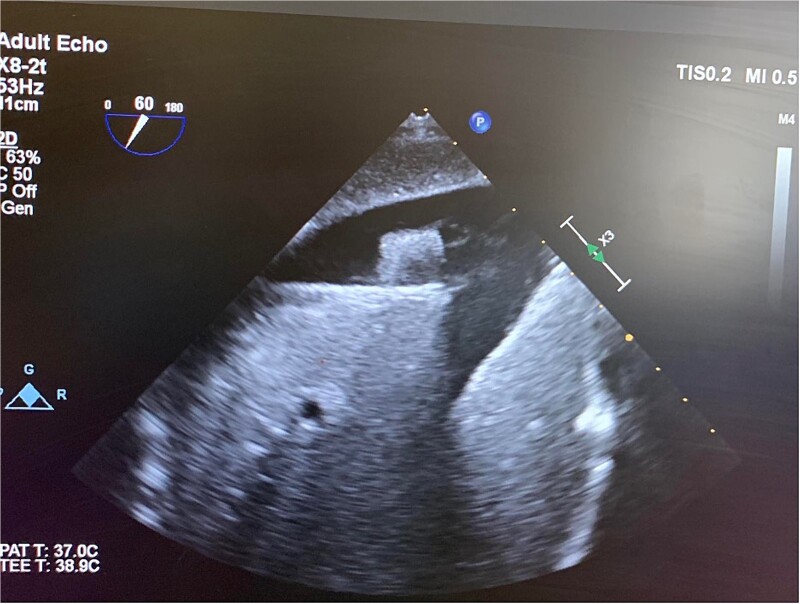
Intraoperative TEE demonstrating the intravascular bullet in the IVC.

In the OR, exploratory laparotomy, right nephrectomy, liver packing, and pericardial window were performed. The pericardial window was negative for blood. With no suspected cardiac injury, intraoperative fluoroscopy (Fig. 1) and transesophageal echocardiogram (TEE) (Fig. 2) were used to locate the bullet. It was suspected to be intravascular secondary to venous embolism from the retro-hepatic inferior vena cava (IVC). Cardiothoracic surgery was consulted for removal of the bullet. With abdominal hemorrhage controlled, the surgeons agreed the patient was an acceptable risk for anticoagulation and cardiopulmonary bypass. Sternotomy was performed, the patient was placed on cardiopulmonary bypass, and the bullet was removed through a right atriotomy.

Postoperatively, the patient was extubated and admitted to the trauma intensive care unit. The patient had an uneventful hospital course. On hospital Day 2, the patient was transferred to a regular nursing floor, and discharged with no sequela on hospital Day 7.

## Case 2

**Figure 3:**
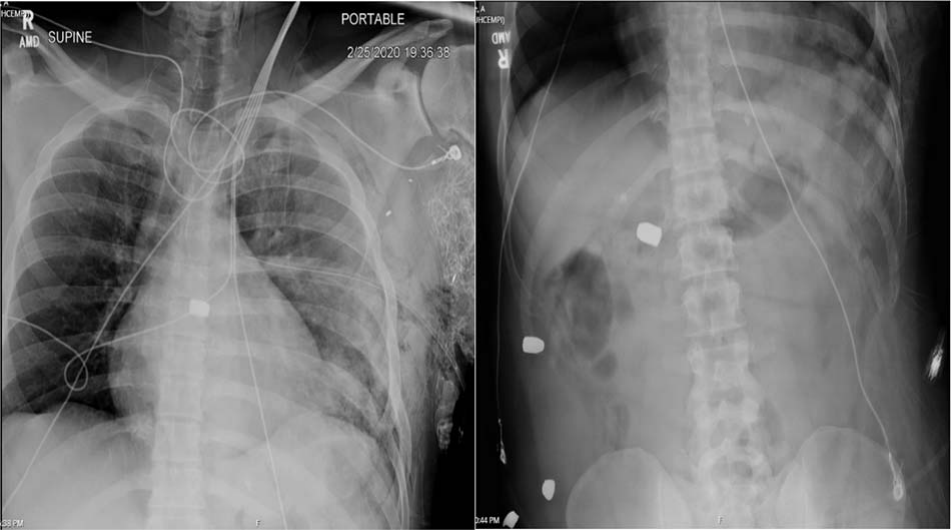
Plain film X-ray obtained in the trauma bay demonstrating multiple bullets overlying the cardiac silhouette and right abdomen.

A 36-year-old male presented with multiple thoracoabdominal GSW (4 GSW in the left axilla, 2 GSW right flank). The patient had decreased breath sounds on the left. Initial vital signs were heart rate 141, blood pressure 70/40, respiratory rate 22, saturation 92% on 15 L non-rebreather, GCS 14. The patient underwent a left thoracostomy tube with 1000 mL initial output and a 9F left femoral central line was placed. The patient was initially resuscitated with 2 units PRBC, 2 units FFP with no improvement in vital signs. A plain film X-ray in the trauma bay revealed multiple thoracoabdominal bullets (Fig. 3). The patient went emergently to the OR for hemorrhage control.

In the OR, left anterolateral thoracotomy and exploratory laparotomy were simultaneously performed. In the thorax, pericardiotomy was performed to release cardiac tamponade, followed by primary repair of a full-thickness left ventricular cardiac injury. Bleeding from the left lower lobe of the lung was controlled by tractotomy and suture ligation. The thoracotomy incision was closed and two 28F chest tubes were placed. In the abdomen, a segmental left colectomy was performed, and temporary abdominal closure placed for a planned second operation. The patient received 15 units PRBC, 12 units FFP, 2 units (5-pack) PLT, 1-unit Cryoprecipitate in the OR. Postoperatively, the patient was admitted to the trauma intensive care unit.

The patient returned to the OR on hospital Day 2 for re-exploration of laparotomy, primary repair diaphragm laceration, TEE. The TEE did not show cardiac shunting between chambers and did not localize the bullet. On hospital Day 3, the patient had a transthoracic echocardiogram which was nondiagnostic and failed to localize the bullet. The patient returned to the OR hospital on Day 3 for re-exploration of laparotomy, colonic anastomosis, and abdominal closure. He was extubated postoperatively and transferred to the regular nursing floor hospital on Day 4.

**Figure 4:**
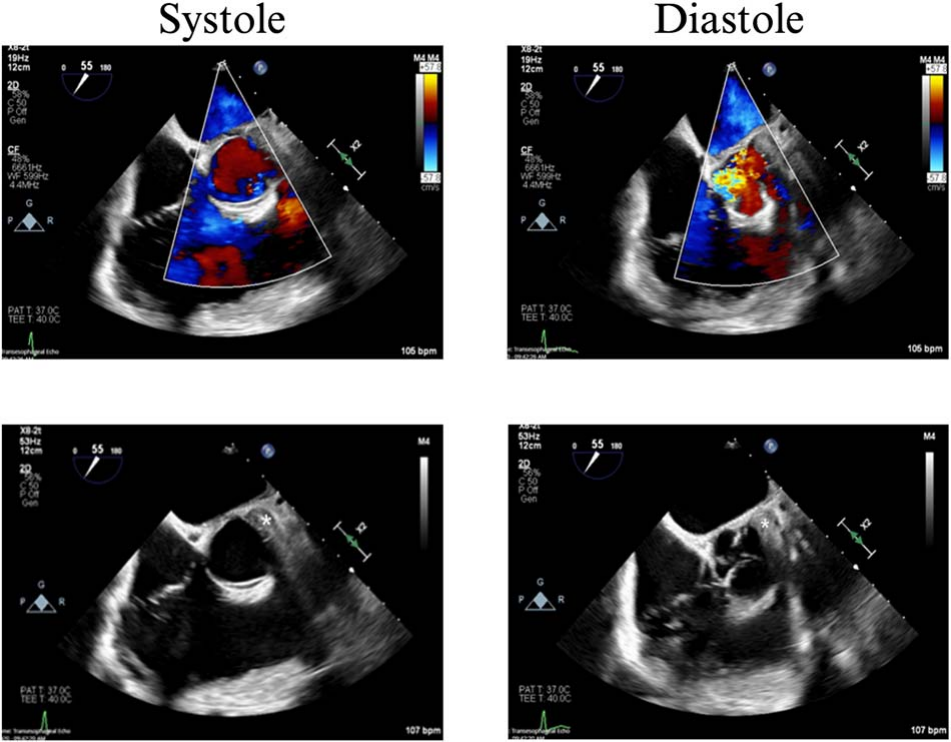
TEE with intracardiac bullet, severe aortic insufficiency. The bullet is labeled by an asterisk, with shadow artifact posterior to the bullet.

On hospital Day 7, the patient had acute hypoxic respiratory failure. CT chest found a right segmental pulmonary embolism and a retained bullet near the aortic root. A TEE was repeated, this time demonstrating severe aortic insufficiency (Fig. 4), and a bullet in the left coronary cusp. Cardiothoracic surgery was consulted for evaluation and management. The next day, the patient went to the OR for median sternotomy, cardiopulmonary bypass, aortic root repair with patch, re-attachment of the left coronary cusp, and removal of the bullet from the aortic root.

Postoperatively, the patient was extubated and recovered in the cardiothoracic intensive care unit. The remainder of the hospital course was uneventful, and the patient discharged home with no sequela on hospital Day 15.

## Discussion

Case 1 involved a stable patient with a single GSW to the left back and a retained bullet over the right ventricle. The trauma team identified the bullet overlying the cardiac silhouette and performed a CT scan to determine its trajectory. The scatter artifact complicated localization, but a pericardial window excluded cardiac injury. Intraoperative imaging (fluoroscopy and TEE) revealed a venous bullet embolism from the retro-hepatic IVC to the right ventricle. The bullet was mobile on fluoroscopy, and TEE provided the best imaging. The trauma and cardiothoracic surgeons discussed bullet removal timing, considering the patient’s stability and heparinization needs. They opted for immediate removal due to the potential lethality of pulmonary artery embolism. The operation was successful, requiring only one surgery for definitive management. This case highlights the success of immediate bullet removal in hemodynamically stable, asymptomatic patients with venous embolism.

Case 2 involved a hemodynamically unstable patient with multiple thoracoabdominal GSWs. The surgical team identified six GSWs and five retained bullets. The patient’s instability precluded CT imaging. A cardiac injury was suspected due to a bullet overlying the cardiac silhouette and significant chest tube output. Thoracotomy confirmed a cardiac injury with tamponade, necessitating immediate repair. The bullet trajectory suggested it passed through the heart and diaphragm into the abdomen. TEE revealed no intracardiac shunting, valve insufficiency, or bullet. The bullet injured the left ventricle and embolized to the aortic root, becoming symptomatic on hospital Day 7. A CT scan suggested an intravascular bullet near the aortic valve, confirmed by TEE showing severe aortic insufficiency. The patient required four operations for definitive management. This case underscores the risks of conservative management and the need for thorough evaluation in unstable patients.

The sensitive nature of various imaging modalities plays a crucial role in locating retained bullets within the body. While screening chest radiography may discover the projectile, it falls short in differentiating among intraparenchymal, intravascular, or intrabronchial locations [8]. The CT scan emerges as a valuable tool in the initial care of stable patients with GSWs, providing insights into hemodynamics and facilitating precise assessment of the bullet’s path and associated damage [9]. Computed tomography exhibits 100% sensitivity, 97% specificity, and 97% accuracy in visualizing cardiac tamponade [10]. TEE is particularly useful for identifying the extent of cardiac damage, ventricular septal defects, or valve injuries, with a sensitivity of 97% for detecting pericardial fluid/tamponade, a specificity of 100%, and an accuracy of 99% [10]. However, scatter infarct from the bullet can complicate the images and make the exact location of these bullets difficult to ascertain. Fluoroscopy-assisted navigational systems offer a rapid and accurate method for urgent removal of metal objects from dangerous locations, minimizing soft-tissue dissection and reducing surgical time and radiation exposure compared to conventional approaches [11]. This innovative technique proves particularly beneficial in complex situations where precise localization of retained bullets near critical structures is essential. We recommend a combination of imaging modalities, including CT scans, transesophageal echocardiography (TEE), and fluoroscopy, which, when combined, prove indispensable in locating bullets both intra- and post-operation, aiding in treatment planning and ensuring optimal patient outcomes.

In these cases, each bullet was ultimately managed via open surgical intervention. However, the use of endovascular techniques for extracting intracardiac missiles has emerged as a feasible option. Yoon et al.’s review of 62 patients with thoracic venous bullet emboli with distributions in various locations: right atrium (9.7%), right ventricle (54.8%), pulmonary arterial tree (32.3%), and intra-thoracic inferior vena cava (3.2%) demonstrated success with endovascular retrieval in 53% of the cases attempted. In instances where endovascular retrieval proved unsuccessful, 28.6% were effectively managed through observation, while 71.4% necessitated open retrieval procedures [1]. These findings underscore the importance of employing a combination of imaging modalities and treatment approaches tailored to the individual patient’s condition for optimal outcomes in managing retained bullets.

We advocate for an individualized and proactive management strategy for all bullet emboli. Identification of GSW/retained bullets should be performed promptly, and bullet trajectories estimated. X-ray and CT imaging help localize retained bullets, and injury to surrounding structures should be assumed. When a discrepancy is found between injury and bullet location, embolism should be considered. TEE and fluoroscopy are intraoperative adjunctive studies that may help diagnose an intravascular bullet. A stable patient with an intravascular bullet should undergo immediate removal. Unstable patients with concerning bullets on X-ray should be stabilized and resuscitated, and life-threatening injuries addressed. When stabilized, a CT scan should be performed to define bullet trajectories and localize retained bullets, especially when multiple trajectories are encountered. Our cases suggest removal of intravascular bullets should be performed when the patient is stable.

## References

[1] Yoon B , Grasso S, Hofmann LJ. Management of bullet emboli to the heart and great vessels. Mil Med 2018;183:e307–13. 10.1093/milmed/usx191. 29659980

[2] Rich NM , Collins GJ, Andersen CA, et al. Missile emboli. J Trauma 1978;18:236–9. 10.1097/00005373-197804000-00002. 660667

[3] Pinto A , Russo A, Reginelli A, et al. Gunshot wounds: ballistics and imaging findings. Semin Ultrasound CT MR 2019;40:25–35. 10.1053/j.sult.2018.10.018. 30686364

[4] Michelassi F , Pietrabissa A, Ferrari M, et al. Bullet emboli to the systemic and venous circulation. Surgery 1990;107:239–45. 2408175

[5] Reese MW , Rendel RE, Collins JN. Management of venous and arterial bullet emboli. Am Surg 2023;89:3614–5. 10.1177/00031348231167413. 36960753

[6] Levi B , Sainsbury CR, Scharf DL. Delayed shotgun pellet migration to the right ventricle. Clin Cardiol 1985;8:367–71. 10.1002/clc.4960080613. 3891182

[7] Shannon FL , McCroskey BL, Moore EE, et al. Venous bullet embolism: rationale for mandatory extraction. J Trauma 1987;27:1118–22. 10.1097/00005373-198710000-00004. 3312620

[8] Sabour AF , Horner L, Douglas G, et al. The pearls and pitfalls of a migrating bullet embolus. Chest 2023;164:e61–3. 10.1016/j.chest.2022.12.051. 37689474

[9] Daghfous A , Bouzaïdi K, Abdelkefi M, et al. Contribution of imaging in the initial management of ballistic trauma. Diagn Interv Imaging 2015;96:45–55. 10.1016/j.diii.2014.02.012. 25540928

[10] Baum GR , Baum JT, Hayward D, et al. Gunshot wounds: ballistics, pathology, and treatment recommendations, with a focus on retained bullets. Orthop Res Rev 2022;14:293–317. 10.2147/ORR.S378278. 36090309 PMC9462949

[11] Mosheiff R , Weil Y, Khoury A, et al. The use of computerized navigation in the treatment of gunshot and shrapnel injury. Comput Aided Surg 2004;9:39–43. 10.3109/10929080400006382. 15792935

